# Facilitators and barriers to the utilization of the ACT SMART Implementation Toolkit in community-based organizations: a qualitative study

**DOI:** 10.1186/s43058-021-00158-1

**Published:** 2021-05-26

**Authors:** Aksheya Sridhar, Amy Drahota, Kiersten Walsworth

**Affiliations:** 1 Department of Psychology, Michigan State University, East Lansing, MI USA; 2 Child & Adolescent Services Research Center, San Diego, CA USA

**Keywords:** Autism, Implementation science, Evidence-based practices, EPIS framework, Web-based, Inner context, Community-based organizations

## Abstract

**Background:**

Evidence-based practices (EBPs) have been shown to improve behavioral and mental health outcomes for children diagnosed with autism spectrum disorder (ASD). Research suggests that the use of these practices in community-based organizations is varied; however, the utilization of implementation guides may bridge the gap between research and practice. The Autism Community Toolkit: Systems to Measure and Adopt Research-Based Treatments (ACT SMART) Implementation Toolkit is a web-based implementation toolkit developed to guide organization-based implementation teams through EBP identification, adoption, implementation, and sustainment in ASD community-based organizations.

**Methods:**

This study examined the facilitators and barriers (collectively termed “determinants”) to the utilization of this toolkit, based on the perspectives of implementation teams at six ASD community-based organizations. Two independent coders utilized the adapted EPIS framework and the Technology Acceptance Model 3 to guide qualitative thematic analyses of semi-structured interviews with implementation teams.

**Results:**

Salient facilitators (e.g., *facilitation teams, facilitation meetings, phase-specific activities*) and barriers (e.g., *website issues*, *perceived lack of ease of use* of the website, *perceived lack of resources*, *inner context factors*) were identified, highlighting key determinants to the utilization of this toolkit. Additionally, frequent determinants and determinants that differed across adapted EPIS phases of the toolkit were noted. Finally, analyses highlighted two themes: (a) Inner Context Determinants to use of the toolkit (e.g., *funding*) and (b) Innovation Determinants (e.g., all website-related factors), indicating an interaction between the two models utilized to guide study analyses.

**Conclusions:**

Findings highlighted several factors that facilitated the utilization of this implementation guide. Additionally, findings identified key areas for improvement for future iterations of the ACT SMART Implementation Toolkit. Importantly, these results may inform the development, refinement, and utilization of implementation guides with the aim of increasing the uptake of EBPs in community-based organizations providing services to children with ASD and their families. Finally, these findings contribute to the implementation science literature by illustrating the joint use of the EPIS framework and Technology Acceptance Model 3 to evaluate the implementation of a web-based toolkit within community-based organizations.

**Supplementary Information:**

The online version contains supplementary material available at 10.1186/s43058-021-00158-1.

Contributions to the literature

- The ACT SMART Implementation Toolkit aims to help community-based organization staff implement evidence-based practices for children with autism spectrum disorder within their organizations.
- Facilitators and barriers to utilizing this toolkit were found within the participating organizations and the toolkit itself. Findings provide valuable information regarding necessary changes for the toolkit in order to facilitate its future use in community-based organizations.
- Results illustrate how two distinct implementation frameworks can be applied together to better understand and utilize web-based implementation toolkits.
- Implementation guides should be developed or revised with end users in mind, such as implementation teams, to facilitate increased utility.

## Background

Autism spectrum disorder (ASD)—a neurodevelopmental disorder affecting 1.8% of the US population [1]—is characterized by deficits in social communication and interactions and the presence of restricted and/or repetitive behaviors, interests, or activities [2]. Use of evidence-based practices (EBPs) developed for this population, including comprehensive treatment models (e.g., Early Start Denver Model) and focused intervention practices (e.g., discrete trial teaching), lead to immediate and long-term improvement in ASD core deficits and social and adaptive functioning [3–6]. However, there is a gap between research and community-based practice use for children with ASD [7–10].

Research indicates that EBP use in community-based organizations (CBOs) varies, such that both EBPs and interventions lacking evidentiary support continue to be delivered [7, 11]. The variable service provision, multiplicity in provider backgrounds and disciplines, and variations in the organizational structure within CBOs result in individuals with ASD receiving fragmented care [12, 13]. Overall, although many autistic children receive their care within CBOs, best-practices are often not utilized in these settings, resulting in limited access to EBPs for this population [14].

Implementation science is an effective way of examining factors, such as “fit” of an existing EBP with the service setting, that impact the adoption and utilization of EBPs in community settings [15, 16]. Implementation frameworks allow researchers to maximize fit by understanding contextual and other factors impacting the compatibility of EBPs and the service setting, resulting in greater facilitation of EBP implementation across settings [17].

**Fig. 1:**
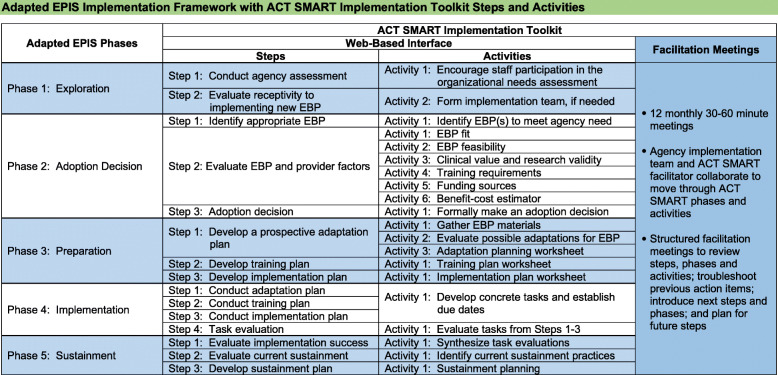
Five systematic phases of the ACT SMART Implementation Toolkit

Moreover, implementation guides can be effective in facilitating EBP implementation across settings by providing a systematic process to enable broader utilization of EBPs within CBOs [17–19]. Based on prior research examining determinants to EBP implementation, the Autism Community Toolkit: Systems to Measure and Adopt Research-Based Treatments (ACT SMART) Implementation Toolkit (hereafter referred to as “the toolkit”) [20] was developed in an effort to address unique client, provider, and contextual factors impacting EBP implementation in CBOs providing services to autistic children and to maximize EBP fit within these agencies [17]. The toolkit is a comprehensive web-based interface guiding organizations through five systematic phases of EBP implementation (Fig. 1). Notably, the toolkit was developed to be agnostic to EBPs; as a result, organizations utilizing the toolkit are able to select an EBP that best fits the needs of their organization from a menu of EBPs developed for this population.

The toolkit was developed in accordance with an adapted version of the Exploration, Preparation, Implementation, Sustainment (EPIS) Framework [21]. Both the original and adapted EPIS are multi-level, multi-step frameworks utilized to understand outer and inner context factors, as well as innovation and bridging factors impacting EBP implementation in community settings [21, 22]. Outer context factors can include service environment and policies, funding, and client characteristics, while inner context factors include organizational and individual characteristics, leadership and staffing within the organization, and self-efficacy, values, and fidelity of providers [17, 23]. Bridging factors highlight the complexity and interaction between outer and inner factors, such as community-academic partnerships [24]. Finally, innovation factors include factors related to the fit of an EBP or innovation within an organization, and characteristics of the EBP or innovation itself [24]. The EPIS Framework has been utilized in numerous studies to examine implementation determinants in public sector settings [14, 24].

Overall, implementation guides, such as the ACT SMART Toolkit, have the potential to bridge the gap between research and practice by addressing implementation barriers and guiding agencies through the implementation process [17–19]. Furthermore, determinant frameworks such as the EPIS allow agencies to identify and address unique factors impacting EBP use within their organization, with the aim of facilitating efficient and effective adoption and implementation of these interventions in CBOs [17]. Pilot study outcomes indicate that the toolkit is feasible, acceptable and useful, and preliminary data suggests that the toolkit is effective (Sridhar & Drahota: Short report: clinical effectiveness of the ACT SMART Implementation Toolkit to facilitate EBP implementation in community-based ASD agencies, in preparation). However, understanding determinants to the toolkits’ use is an integral step to enable its broader adoption and positive impact in community settings. Therefore, we explore the following:

- What are the facilitators and barriers to the utilization of the toolkit as reported by agency implementation teams?
- Did the identified determinants differ by adapted EPIS phase?

## Method

Study data was collected as part of a larger pilot evaluating implementation outcomes and fidelity of the toolkit with a small sample of ASD-CBOs in Southern California. Procedures were approved by appropriate institutional review boards; the secondary data analysis involved in the current study was approved through the Michigan State University IRB. Authors followed the Standards for Reporting Qualitative Research (SRQR) [25] to increase the quality and transparency of this research.

### Design

This study involved qualitative data analysis of semi-structured interviews with participating ASD-CBOs.

### Frameworks

Two determinant frameworks guided qualitative data analysis (i.e., coding, thematic analysis). The adapted EPIS Framework [17], which also guided toolkit development, allowed for analysis of contextual and innovation determinants to toolkit use. Additionally, this study utilized the Technology Acceptance Model (TAM3; [26]), the most widely used model in research examining adoption of technology products. The TAM3 examines the perceived ease of use (extent to which staff believe the technology will require no effort to use) and usefulness (extent to which staff believe the technology will enhance job performance) of a product and determinants (e.g., system characteristics, facilitating conditions) that may impact these perceptions [26–29]. In this study, the TAM3 guided analysis of determinants and the “perceived ease of use” and “perceived usefulness” specific to the technological components of the toolkit (e.g., website). Neither framework was utilized in data collection procedures.

### Context

Six ASD-CBOs located in urban areas in Southern California participated in the pilot study. Organizations were selected based on (1) existing social and/or research collaborations with other agencies, researchers, or collaborative groups; (2) existing efforts to receive additional training for their staff; and (3) interest in implementing new EBPs within their agency.

### Participants

Participating ASD-CBOs (Table 1) identified a need for one of the three ASD-EBPs selected for the pilot study: social narratives (describing social situations, highlighting important social cues, and offering examples of appropriate responses), video modeling (observing successful engagement of a specific behavior to encourage imitation), or self-management (monitoring one’s self to independently regulate their own behaviors in a variety of situations). These EBPs were selected as they are focused EBPs rather than comprehensive treatment packages [3], and none had specific training or certification requirements [30]. Additionally, robust evidence for the efficacy or effectiveness exists for the interventions [3].

**Table 1:** Participating ASD-CBOs

ID	Services provided	ASD-CBO maturity	Service setting	Number of clients	% ASD clients	Age range served
**1**	Applied behavior analysis	10 years	Home, community	99	60%	0–5 years: 5%
						6–10 years: 25%
						11–15 years: 30%
						16–18 years: 15%
						18+ years: 25%
**2**	Applied behavior analysis	1 year, 6 months	Home, community	40	99%	0–5 years: 70%
						6–10 years: 25%
						11–15 years: 0%
						16–18 years: 3%
						18+ years: 2%
**3**	Applied behavior analysis	3 years	Home, community, school	50	100%	0–5 years: 95%
						6–10 years: 5%
						11–15 years: 0%
						16–18 years: 0%
						18+ years: 0%
**4**	Speech and language pathology	12 years, 2 months	Clinic, community, school, home	350	50%	0–5 years: 52%
						6–10 years: 40%
						11–15 years: 4%
						16–18 years: 2%
						18+ years: 2%
**5**	Applied behavior analysis and mental health	5 years, 3 months	Clinic	400	60%	0–5 years: 30%
						6–10 years: 45%
						11–15 years: 20%
						16–18 years: 5%
						18+ years: 0%
**6**	Applied behavior analysis	18 years	Home, community, school	170	80%	0–5 years: 43%
						6–10 years: 32%
						11–15 years: 12%
						16–18 years: 3%
						18+ years: 10%

Five ASD-CBOs selected video modeling as the EBP to be implemented within their organization and completed all phases of the implementation process using the toolkit. One organization chose not to adopt an EBP at the end of phase 2 (*Adoption Decision Phase*) due to a lack of fit between agency needs and the EBPs. Implementation teams (ITs, *N* = 6) at each organization were made up of a range from 1 to 4 staff members and included agency leaders, supervisors, and direct providers (Table 2).

**Table 2:** IT demographics

Demographics	Agency leaders (*n* = 7)	Supervisors (*n* = 8)	Direct providers (*n* = 1)
**Sex (female)**	100%	100%	100%
**Race/ethnicity**			
White	100%	25%	100%
Mixed race/ethnicity	–	25%	–
Prefer not to answer	–	12.5%	–
**Education level**			
Master’s degree	42.9%	50%	100%
Doctorate	57.1%	12.5%	–
**Educational discipline**			
Psychology	28.6%	25%	–
Behavior specialist	28.6%	25%	100%
Speech/language/communication	28.6%	12.5%	–
Education	14.3%	–	–
**Missing** (37%, supervisors)			

ITs were required to have at least one team leader. Agency leaders were eligible if they (a) had a role of director, CEO, or lead decision-maker regarding treatment use at their organization; (b) were willing to commit 1 year of study engagement; and (c) agreed to provide feedback following each phase and at the end of the pilot. The remainder of each IT was made up of organizational staff members, as determined by the IT agency leader. All IT participants consented to participate and to provide feedback about the toolkit.

### Procedure

Semi-structured interviews were conducted with each participating IT after each phase was completed and at the end of the pilot study. Interviews with ITs were audio-recorded and conducted in person at a location convenient to the members or by phone. Interviews began with questions using a Likert Scale format (this data was not utilized in the current study), and responses were followed up with open-ended questions about why a score was given, areas for improvement, and factors impacting the respondent’s perceptions. If the interview involved more than 1 person, then each individual was asked to provide a response. ITs were compensated $100 at the end of each interview. End of phase interviews lasted 10.12 min (*SD* = 4.32) and end of pilot interviews lasted 22.79 min (*SD* = 6.86).

### Measures

#### End-of-phase interviews

End-of-phase interviews (Additional file 1) focused on perspectives regarding the feasibility, acceptability, and utility of the activities and facilitation meetings that occurred throughout that specific phase. Additionally, ITs were asked to report any changes that had been observed at their organization from the beginning of the study to the date of the interview and recommendations for revisions to any component of the phase.

#### End-of-pilot interviews

Post-pilot interviews (Additional file 2) focused on the feasibility, acceptability, and utility of the toolkit and facilitation meetings overall. IT members were also asked about challenges their organization faced while using the toolkit, perspectives on the toolkit and facilitation meetings, views on the impact of the toolkit in their organization (i.e., success, value), future interest in using the toolkit, and revision recommendations.

### Data analysis

#### Data processing

Recorded interviews were transcribed and verified by research assistants unfamiliar with the aims of the pilot or current study. Interview data were anonymized, and, in cases of multiple respondents, each respondent was randomly assigned a number (e.g., participant 1, participant 2) for each transcription. Although each IT was made up of a range of 1–4 members, not all members were present at every interview. Data was processed utilizing each IT as one unit of analysis, rather than analyzing each participant within the IT as separate analysis units [31].

#### Thematic analysis

Thematic analysis [32] was conducted to explore facilitators and barriers to the utilization of the toolkit, by phase and after 1 year, through the development of relevant main codes, subcodes, and themes across the entire data set. The analysis was conducted by two independent coders, and researcher characteristics and reflexivity (i.e., common biases and assumptions related to this research) were considered and discussed by both coders, in accordance with the SRQR [25].

#### Coding

A number of coding methods were utilized [33], including provisional coding (i.e., codes developed a priori based on research questions and implementation frameworks). For example, the code “Perceived ease of use” was identified a priori based on the TAM3, and deductive coding captured interview responses such as “[the website] didn’t seem user friendly.” Line-by-line open coding was utilized to identify emergent codes [34]; for example, in alignment with the EPIS framework, “funding” emerged through inductive coding, and fell under “Inner Context Factors” (an EPIS construct identified a priori). During this process, a codebook (Additional file 3) was developed and regularly revised. An audit trail tracked this iterative process. Thereafter, subcoding was utilized to identify second-order codes nested under primary codes. This provided greater detail about main codes (e.g., Main code: Barriers to the use of the toolkit; Subcode: “Website Issues”). Finally, axial coding was used to group similar codes together. Once the codebook was complete, the independent coders conducted a final coding of all interviews to ensure coding consistency across the data. Throughout, consensus coding was used to handle discrepancies. MAXQDA, a qualitative and mixed methods computer software, was used to examine coding frequencies and to facilitate the review of illustrative quotes by main code, subcode, and theme.

Salient codes were identified based on the independent coders’ perspectives regarding codes believed to be most impactful in either facilitating or hindering the use of the toolkit. In addition, the frequency of the codes across all transcripts was considered to support the saliency of codes [35].

#### Theming the data

The coders developed two main themes that aligned with the guiding frameworks, following the process outlined by Braun & Clarke [36]. Using MAXQDA, the coders examined each code to determine whether the content of these codes differed by adapted EPIS phase.

## Results

### Frequent codes

Qualitative data was quantized to determine frequency counts (e.g., number of times the code was assigned across all interview transcripts). *Phase-specific activities* (subcode: *as a facilitator*) to the utilization of the toolkit was coded most frequently, followed by *facilitation team (FT) meetings*, and *phase-specific activities* (subcode: *as a barrier)*. Frequencies, descriptions, and illustrations of each code, organized by theme, are reported in Table 3.

**Table 3:**
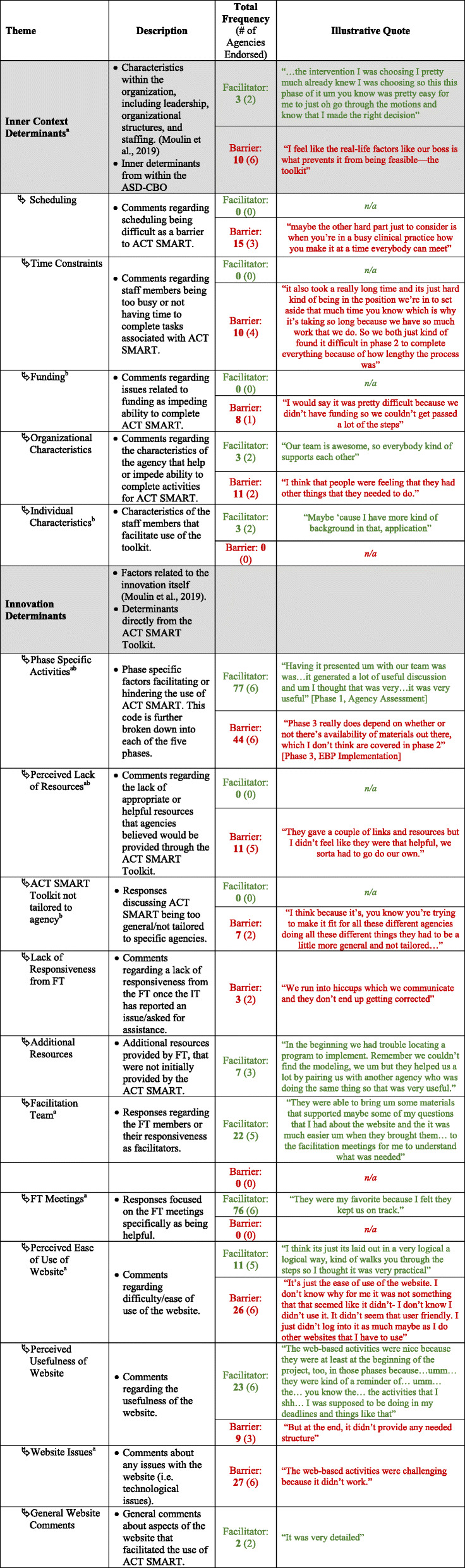
Themes, descriptions, frequencies, and illustrative quotes

^a^Salient determinants

^b^Determinants that differed across phases

### Salient codes

#### Salient facilitators

Three salient facilitators were identified and contextualized within the implementation frameworks; all three aligned with the EPIS Frameworks’ “innovation factors” [24]. Firstly, ITs discussed supportive characteristics of the *facilitation teams* (FTs) that facilitated ITs use of the toolkit, including being responsive to agency needs, providing support to IT members, and being flexible when scheduling and meeting. One IT member stated, “[facilitator’s] been really communicative and we haven’t run into anything where we felt like we couldn’t get support.” Secondly, FT meetings were often discussed as a facilitator to toolkit use, with one IT member commenting: “they were very, very helpful. I would say they were probably one of the most helpful […] aspects of the ACT SMART.” IT members cited several reasons for FT meetings being helpful, including that they helped ITs “*stay on track*,” provided accountability, allowed IT members to ask clarifying questions, and provided helpful information as teams proceeded through the toolkit.

Finally, *phase-specific activities* were identified as a salient facilitator to toolkit use. In particular, activities from phases 1 (*Exploration*) and 2 (*Adoption Decision*) were often discussed by ITs. The agency assessment and ACT SMART orientation meeting (phase 1) were described as being particularly helpful, as they provided ITs with valuable information regarding agency needs, current resources at the agency, and about the toolkit itself. In phase 2, the cost estimator worksheet was reported as helpful to ITs when they considered the cost of implementing the EBP, including staffing, resources, and training costs. IT members discussed needing this information to move forward in utilizing the toolkit and prepare for EBP implementation. As stated by one IT member, “I think the cost benefit analysis is useful and […] necessary.” Overall, facilitation teams, FT meetings, and phase-specific activities appeared to be most salient for supporting IT use of the toolkit across all phases, as well as facilitating EBP implementation preparation.

#### Salient barriers

Four salient barriers were identified. *Website issues*, described as “a couple glitches that need to be […] worked out” was selected as a salient code based on IT responses regarding numerous technological issues when using the website. This was especially significant given that the majority of toolkit activities were intended to be completed on the website; as a result, technological issues often made it difficult for agencies to complete web-based activities. This code aligned with the adapted EPIS framework as an innovation factor, as it was specific to the toolkit website.

Secondly, the *perceived ease of use* of the website was identified as a salient barrier to toolkit use. IT members discussed difficulty navigating the website, stating “it was very hard to navigate” and “it wasn’t […] user-friendly.” These difficulties led several IT members to avoid use of the website to complete activities during the pilot study. This code aligned with the TAM3, which highlights perceived ease of use as a determinant for adoption and use of technology products during the implementation of innovations in community settings [26].

Thirdly, IT members discussed their *perceived lack of access to resources* they believed would have been helpful. For example, IT members expressed a need for access to behavior analytic journals specifically, and the ability to communicate with other agencies that had implemented the same EBP to discuss implementation with agencies who underwent a similar process. Finally, agencies reported a *perceived lack of resources* related to the chosen EBP as a barrier to EBP implementation, as well as hindering implementation teams’ ability to progress through toolkit phases. One IT agency leader stated “so […] we sort of had to do our own research, identify our own materials […] when we were gathering the materials together [for video modeling].” The *perceived lack of resources* code aligned with the EPIS framework, highlighting the importance of accessing resources when implementing EBPs in community settings [24]. This code fell under the EPIS frameworks’ “innovation factors,” as IT members believed these resources would be provided through the toolkit.

Finally, *inner context factors* emerged as a salient code and included issues related to scheduling and funding within the organization. Specifically, IT members discussed staff being very busy, leading to difficulties in scheduling meetings focused on completing toolkit activities. Additionally, IT members discussed difficulty accessing resources needed for EBP implementation due to a lack of funding within the organization. Both barriers align with the EPIS framework’s “inner context factors” [24].

### Thematic analysis findings

**Fig. 2:**
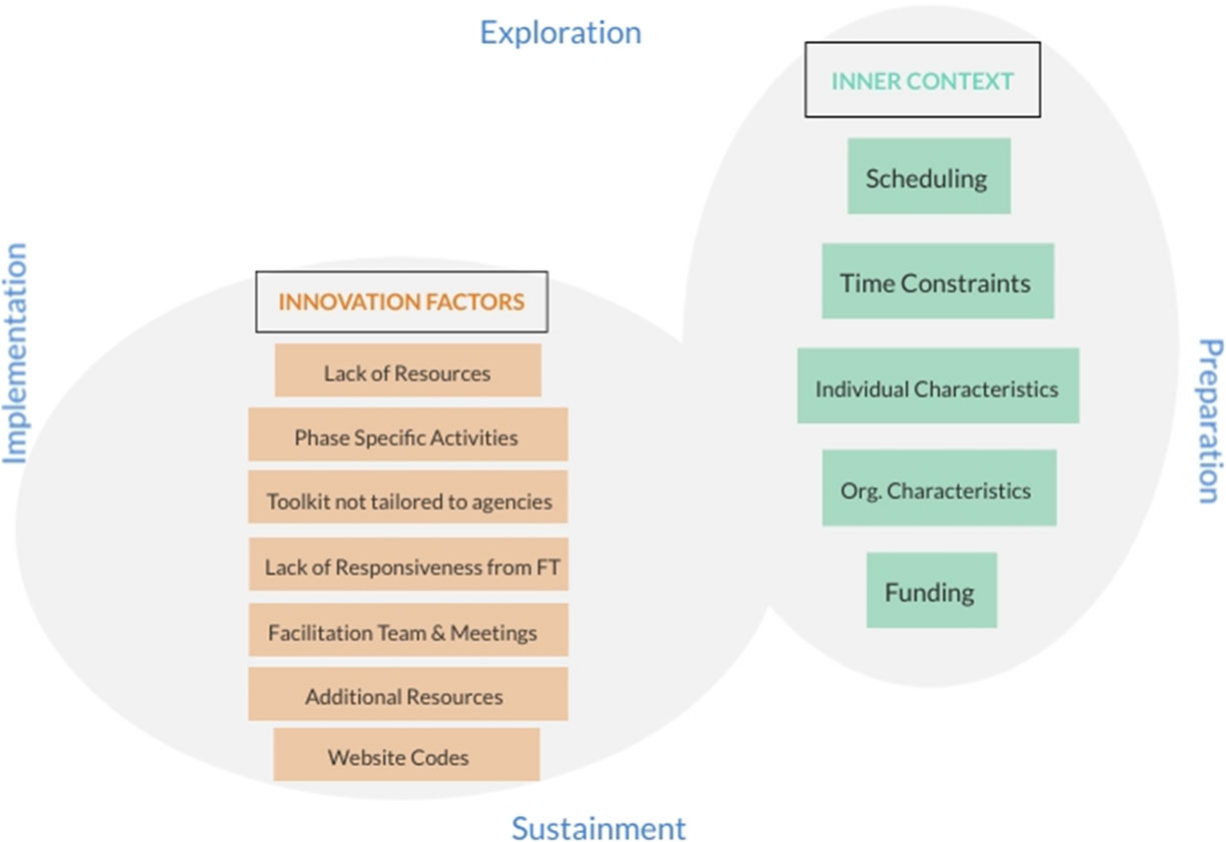
Two themes developed utilizing both the EPIS framework and TAM3: Inner Context Determinants and Innovation Determinants

Finally, two themes were developed utilizing both the EPIS framework and TAM3: *Inner Context Determinants* and *Innovation Determinants* (Fig. 2). Coders grouped each code under one of the two themes and then categorized each code as a facilitator, barrier, or both, depending on the IT members’ responses. Finally, researchers utilized MAZQDA to determine whether the content of each code varied by adapted EPIS phase.

#### Inner context determinants

**Fig. 3:**
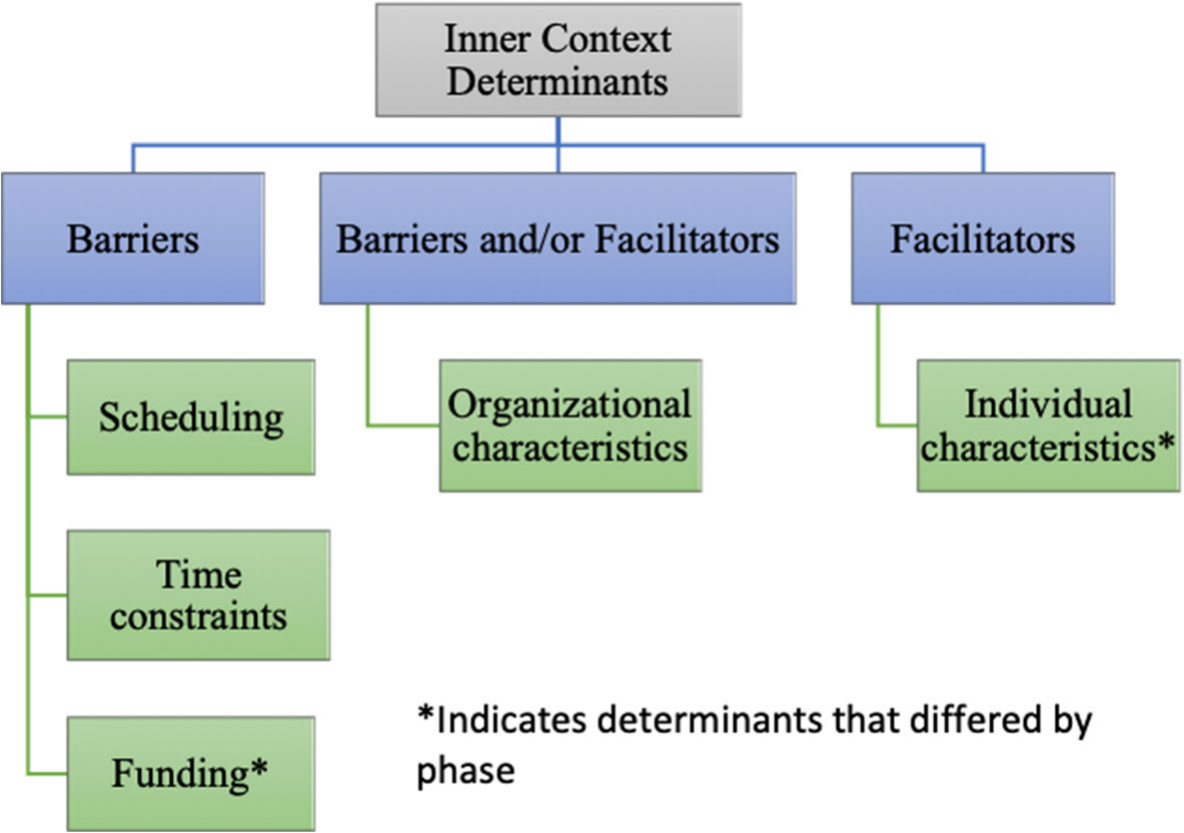
Inner Context Determinants to toolkit's use

This theme highlighted a number of factors within the participating CBOs that were determinants to the toolkit’s use (Fig. 3) and aligned with the adapted EPIS Framework [17, 23]. *Organizational characteristics*, such as staff buy-in from numerous members of the organization, facilitated the use of the toolkit (e.g., “we’ve got a really good staff who was […] very eager to do it”). However, *organizational characteristics* also acted as a barrier. For example, IT members perceived their organizations to have other priorities over the toolkit, making it difficult for IT members to complete toolkit activities. Additional inner-contextual facilitators included *individual-level* (i.e., staff) *characteristics*, such as “having that [staff training] background,” while barriers to the utilization of the toolkit included *funding*, *time constraints*, and *scheduling*.

#### Inner context determinants that differed by phase

Two “Inner Context Determinants” differed by adapted EPIS phase. Facilitating *individual-level characteristics* differed across phases such that prior experience and familiarity with leading staff trainings was an important facilitator for two organizations in phase 4 (*Implementation*), when staff training occurred; this was not discussed within other phases. For example, during an end-of-pilot interview, an IT member explained, “there was times that [staff member] was really key in the implementation, because she was leading the training.” *Funding* was a barrier across all phases for one organization and was particularly salient in phase 3 (*Preparation*), when agencies were purchasing materials and resources needed for implementing the EBP. Lack of funding at this organization prevented the ITs ability to purchase materials during this phase. Notably, *funding* was not discussed by any other organization during any phase of the toolkit. No other Inner Context determinants differed by phase.

#### Innovation determinants

**Fig. 4:**
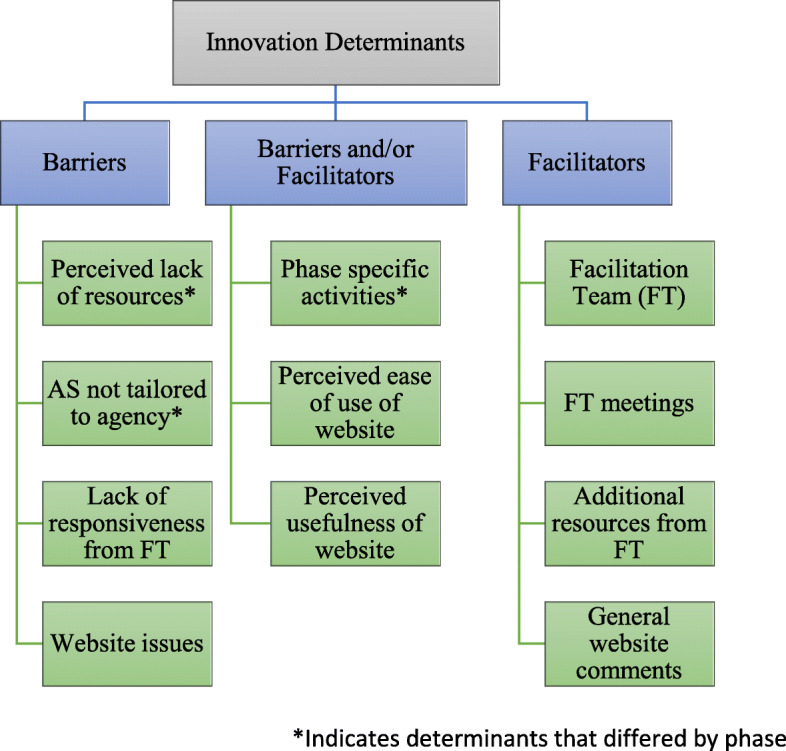
Innovation Determinants to toolkit's use

The second theme, “Innovation Determinants,” highlighted factors specific to the toolkit as salient determinants to its use [24]. Codes under this theme included any component that specifically came from the toolkit, such as *facilitation teams and meetings*, *phase-specific activities* developed for the toolkit, and all comments related to the *ACT SMART Website* (Fig. 4) that facilitated or hindered toolkit use.

#### Innovation determinants that differed by phase

Although no innovation facilitators were found to differ by phase, three innovation barriers differed by adapted EPIS phase, including phase-specific activities. Firstly, the toolkit was described by respondents as *not tailored to specific agencies*; this was particularly notable in phases 2 (*Adoption Decision*) and 4 (*Implementation*). For example, one organization expressed a need for behavior analytic journals, specifically, to match their needs as an organization providing ABA to clients, while a SLP organization explained that “it was just difficult for us, it-it wasn’t difficult it was just challenging for us to adapt s-some of these materials to our particular population or therapy targets.” Additionally, a general *perceived lack of resources* during phases 2 (*Adoption Decision*) and 3 (*Preparation*) emerged from the data, as IT members described needing specific materials for the chosen EBP and had difficulty accessing these resources (e.g., training videos; training manuals). Overall, these innovation barriers were salient to implementation teams throughout toolkit phases 2 to 4.

#### Phase-specific activities as innovation determinants

A number of *phase-specific activities* were identified as facilitators and/or barriers that differed by phase. Specifically, the agency assessment in phase 1 (*Exploration*) was reported to facilitate the use of the toolkit by some respondents. For example, one IT member stated, “the useful part is getting the data” and explained that the agency assessment allowed agencies to gather information on training needs and resources. However, this activity was also described as “confusing” and “time-consuming;” therefore, some IT members felt this activity hindered the utilization of the toolkit during this phase. Similarly, the cost estimator worksheet (phase 2, *Adoption Decision*) was helpful in facilitating the development of a budget for EBP implementation within each agency. As stated by one IT member, “it does help you plan out and also helps you to anticipate […] what the funding is gonna be.” However, this activity presented logistical barriers because “there was the glitch, it didn’t work” on the website, and the worksheet was described as “*time-consuming*” or “*confusing*” by different IT members. Finally, phase 3 (*Planning*) included an implementation strategy activity and development of a staff training plan that were perceived as being helpful to the agencies and facilitated the utilization of the toolkit during this phase, specifically. Overall, activities in phases 1 through 3 were particularly salient to implementation teams. No other innovation barriers or facilitators were found to differ by phase.

#### Website factors as innovation determinants

Importantly, a number of codes under the Innovation Determinants theme were specific to the web-based component of the toolkit. These codes were not found to differ by phase but were important to highlight as the website made up a significant portion of the toolkit. Two of these codes aligned with the TAM3 [26]: the *perceived ease of use* (e.g., “*really user friendly*”) and *perceived usefulness* (e.g., “*not useful, we didn’t use it*”) of the website. These were identified as either facilitators or barriers to the utilization of the toolkit and highlight the importance of IT members’ perceptions of the utility and feasibility of using a technological product to increase EBP uptake at their agencies. In addition, two codes related to other comments regarding the toolkit website were identified: *website issues* were a barrier (e.g., “*glitches with the website*”) and *general comments regarding the website* (e.g., “*it did not take that much time*”). While these codes did not align with the TAM3 specifically, they highlight other important factors to consider when using a technological product to support the application of implementation strategies in CBOs.

## Discussion

This study aimed to identify determinants to the utilization of the ACT SMART Implementation Toolkit and to explore whether these determinants differed by implementation phase. The results highlighted several salient facilitators and barriers to toolkit use. Two themes that aligned with both the EPIS Framework and the TAM3 were identified to conceptualize these determinants. Results indicate an interaction between the two implementation frameworks.

### Salient determinants

Salient facilitators to toolkit use included *facilitation teams*, *facilitation meetings*, and *phase-specific activities*. These findings highlight the importance of facilitation as a component of the toolkit; IT members consistently described this aspect of the toolkit as being the most helpful in facilitating the implementation of the selected EBP. Additionally, phase-specific activities included in the toolkit were found to provide agencies with helpful and necessary information as they progressed through the adapted EPIS phases.

Salient barriers included *website issues*, *perceived lack of ease of use* of the website, *perceived lack of resources*, and *inner context factors*. These barriers suggest that while the website provided helpful resources or was otherwise useful to the participating agencies, technological problems (e.g., “glitches”) and difficulty navigating the website led most ITs to avoid using the website to complete toolkit activities during the pilot study. Furthermore, these findings highlight the need for future iterations of the toolkit to address these technological problems and focus on making the website more user-friendly and easier to navigate. Additionally, IT members believed that the toolkit would be responsible for providing certain resources (e.g., access to journals, EBP training videos); however, the toolkit did not include these resources, as it was not developed for specific service types or EBPs. As a result, this barrier appeared to stem from a possible misunderstanding regarding the resources included as part of the ACT SMART Implementation Toolkit. Future use of this toolkit should ensure clear communication regarding the resources provided by the toolkit, as well as resources the agencies are responsible for accessing (e.g., resources for the chosen EBP). Finally, Inner Context Factors, such as *scheduling* and *funding*, seemed to hinder the use of the toolkit, such that ITs were unable to progress to a subsequent phase if lacking adequate funding or due to the inability to schedule time to complete toolkit activities. Overall, these findings suggest that inner context organizational factors continued to hinder EBP implementation in ASD-CBOs, despite utilizing a systematic and structured implementation toolkit, highlighting a need for further research on addressing inner contextual barriers to implementation.

### Determinants across implementation phases

Several determinants differed across implementation phases. Of note, phase-specific activities differed by phase and were perceived as both facilitators and barriers. For example, both the agency assessment (phase 1) and the cost estimator worksheet (phase 2) provided agencies with helpful information needed prior to EBP adoption; however, both activities were also described as being time-consuming and confusing to complete. Importantly, these findings indicate an interaction between various innovation factors, such that the content of the activities facilitated toolkit use, while the modality in which these activities were delivered impeded toolkit use. These findings are an important contribution to understanding how determinants may interact to facilitate or hinder the implementation of innovations within CBOs.

These findings also suggest that determinants may have differed by phase based on the purpose or goals of each specific phase. For example, in phase 2 (*Adoption Decision*), barriers such as the *perceived lack of resources* and the *ACT SMART Toolkit not being tailored to agency needs* appeared to be salient to IT members. Based on IT member responses, these factors played a significant role during adoption decision-making due to the need for specific and tailored resources that matched agency needs. In phase 3 (*Preparation*), the *perceived lack of resources* and lack of *funding* were found to be the greatest barriers to use, as agencies began to purchase resources and materials needed for the implementation phase. Finally, in phase 4 (*Implementation*), *individual-level characteristics*, such as prior experience leading staff trainings, was found to be a significant facilitator, as agencies began to implement training plans prior to EBP implementation. Overall, these findings indicate that a number of determinants were particularly significant depending on the purpose and activities during specific phases of the toolkit. Importantly, a number of determinants (e.g., time constraints, staff buy-in) were found to be present across all phases of implementation, indicating that some determinants played an important role throughout the toolkit’s use.

### Contribution to implementation science

These results advance the field of implementation science in two notable ways: contributing to the implementation science framework literature and providing guidance related to the development of implementation guides. First, these results advance our understanding and use of implementation frameworks. Specifically, these results highlight inner context and innovation determinants to the utilization of the toolkit. Findings support previous research indicating that inner context factors such as *funding* and *staff buy-in* are important determinants to the implementation of innovations in community settings [23, 37, 38]. Findings also illustrate additional inner context determinants not previously discussed in the literature, such as *scheduling, time constraints,* and *individual provider characteristics*, suggesting areas for future research. Additionally, these findings highlight several aspects of the complex innovation that acted as determinants to the use of the toolkit. Specifically, *facilitation team* and *facilitation team meetings* were perceived to be one of, if not the most, helpful aspects of the toolkit. This finding aligns with previous literature illustrating the importance of facilitation in the implementation of interventions across settings [39]. Additionally, findings support previous literature indicating that a perceived lack of resources for specific EBPs is often a barrier to implementation in community-based settings [10, 40]. Finally, both the *perceived ease of use* and the *perceived usefulness of the website* were found to be important to the utilization of the toolkit. This is consistent with previous literature suggesting these two factors are highly predictive of technology use in various settings [26]. Importantly, these findings indicate an interaction between both implementation frameworks, such that salient determinants within the TAM3 were found to fall under the “innovation factors” component of the EPIS. This suggests that these frameworks may be utilized in tandem to understand determinants to the implementation of technology-based products as an innovation in CBOs. Notably, this study is the first to utilize both the TAM3 and the EPIS framework jointly and to highlight ways in which these implementation frameworks may interact.

In addition, this study contributes to the understanding of utilizing implementation guides within CBOs. Implementation guides are hypothesized to help facilitate an organization’s EBP adoption, uptake, implementation, and sustainment [18, 19]. Similar to evidence indicating that EBP characteristics influence an EBP’s adoption and utilization, these findings suggest that the characteristics of an implementation toolkit may facilitate or hinder their use, such as innovation fit (e.g., with the system, organization, and implementation team), ease of use, relative advantage, trialability, observability, and low complexity [16, 24, 41]. Developers must design (or revise) implementation guides with the end user in mind, such as organizational implementation teams, to ensure that the benefit from a systematized implementation approach can be realized.

## Limitations

Due to the small sample size of the pilot study, generalizability of this data is limited. Furthermore, although six agencies took part in the pilot study, only five agencies completed all phases of the toolkit. Interviews were conducted with the agency that chose not to implement an EBP; however, this agency only completed two phases of the pilot study, limiting the amount of data available during analysis. Additionally, due to the small sample size and limited time frame of the pilot study, thematic saturation was unable to be established. Overall, future studies examining the utilization of implementation guides should aim to include a larger sample size, when possible. Importantly, two interview recordings (agency 1: end-of-phase 4 and end-of-pilot) were missing from analysis, further limiting the amount of data available for analysis. Additionally, although interviewers were trained to elicit responses from all implementation team members involved in the interviews, it is possible that power dynamics among agency staff (e.g., agency leaders and staff) influenced responses. Future research may address this limitation with the use of individual interviews or by providing respondents the opportunity to follow-up with interviewers individually. As well, this study involved secondary data analysis collected in a different state. As a result, researchers did not have the ability to conduct data checking with implementation teams at the participating agencies following qualitative analysis. It is possible that the lack of data checking may have led to biased interpretations of interview data. However, by utilizing the methods outlined as part of the SRQR [25], the researchers aimed to mitigate the impact of their biases on this qualitative analysis.

With regard to the implementation frameworks utilized, it is important to note that coders were not able to examine factors purported to impact *perceived usefulness* and *perceived ease of use* within the TAM3. The TAM3 posit that factors such as individual differences, system characteristics, social influence, and facilitating conditions influence perceptions regarding ease of use and usefulness of a technology product. However, these factors were not explored during the interviews. Future research utilizing technology products within an implementation guide should aim to explore factors impacting perceptions of the technology product.

## Conclusion

This study illustrates frequent and salient facilitators and barriers to the utilization of the ACT SMART Implementation Toolkit that was designed to be used within ASD-CBOs. Specifically, this study identified several salient facilitators (i.e., *facilitation teams*, *phase-specific activities*) that should be included in future iterations of the toolkit, as well as significant barriers to the utilization of this toolkit (i.e., *website issues*, *time constraints*) that should be addressed in toolkit revisions. Although these findings are specific to the ACT SMART Implementation Toolkit, a number of the factors identified as facilitators and barriers in the present study are consistent with previous studies that have illustrated similar determinants to the implementation of innovations in community-based settings [27, 38–40]. Overall, these findings illustrate areas for improving the toolkit that may be addressed in future studies, with the ultimate aim of increasing the uptake of EBPs in CBOs providing services to children with ASD. Finally, this paper is the first to utilize the EPIS Framework and Technology Acceptance Model 3 in tandem and highlights how these frameworks may be utilized together in future research to understand factors impacting the implementation of technology-based innovations in CBOs. Future research may utilize these findings to inform the development or improvement of other implementation guides utilized in CBOs or other web-based implementation toolkits.

## Supplementary Information

**Additional file 1.** End of Phase Interview Guide.

**Additional file 2.** End of Pilot Interview Guide.

**Additional file 3.** Codebook.

## Data Availability

The datasets used and/or analyzed during the current study are available from the corresponding author on reasonable request.

## Abbreviations

ASD: Autism spectrum disorder
EBP: Evidence-based practice
CBO: Community-based organization
ASD-CBO: Community-based organization for individuals with autism spectrum disorder
ACT SMART: Autism Community Toolkit: Systems to Measure and Adopt Research-Based Treatments
EPIS: Exploration, Preparation, Implementation, Sustainment framework
TAM3: Technology Acceptance Model 3
SRQR: Standards for Reporting Qualitative Research
ABA: Applied behavior analysis
SLP: Speech and language pathology
IT: Implementation team
FT: Facilitation team
