# Supplementary material for: Facilitators and barriers to the utilization of the ACT SMART Implementation Toolkit in community-based organizations: a qualitative study
Source: Implement Sci Commun. 2021 May 26;2:55. doi: 10.1186/s43058-021-00158-1 (PMC8157454; doi:10.1186/s43058-021-00158-1)
Supplement: Supplementary file 1 — Additional file 1. End of Phase Interview Guide. [file 43058_2021_158_MOESM1_ESM.doc]

## Additional File 1. End of Phase Interview Guide

**ACT SMART End of Phase Interview Guide**

I will be asking you a few questions today about your perceptions of the feasibility, acceptability, and usefulness of phase _______ of the ACT SMART toolkit.

A. ACT SMART Toolkit

1. How practical was it to complete phase ____ of the ACT SMART toolkit? By Phase ____, I mean reading the website content, completing activities, and how the website functioned.

| **1** | **2** | **3** | **4** | **5** |
| --- | --- | --- | --- | --- |
| Not at all | Slightly | Moderately | Very | Extremely |

1. Why did you give it that score?

(*Optional: What would need to be different to give it a higher score? Was there anything that wasn’t practical or could be improved?)*

1. How useful was this phase of the ACT SMART toolkit? Again, I mean reading the website content, completing activities, and how the website functioned.

| **1** | **2** | **3** | **4** | **5** |
| --- | --- | --- | --- | --- |
| Not at all | Slightly | Moderately | Very | Extremely |

1. Why did you give it that score?

*(Optional: What would need to be different to make this phase more useful? Was there anything that wasn’t useful or could be improved?)*

1. How satisfied were you with this phase of the ACT SMART toolkit?

| **1** | **2** | **3** | **4** | **5** |
| --- | --- | --- | --- | --- |
| Strongly Dissatisfied | Dissatisfied | Neutral | Satisfied | Strongly Satisfied |

1. Why did you give that score?

*(Optional: Is there anything that can be done to improve your satisfaction score? Why do you say that? Can you tell me why that is?)*

B. ACT SMART Training Model

1. What did you think about the facilitation meetings that took place during this phase?

*(What made you satisfied with them? What made them practical or useful?)*

C. Impact of ACT SMART on agency

1. What kind of changes have you seen at your agency since the beginning of ACT SMART?

*(Optional: None, why is that?)*

D. Recommendations

1. Are there any changes that you would recommend for the toolkit and the facilitation meetings for this phase?
