# Supplementary material for: Facilitators and barriers to the utilization of the ACT SMART Implementation Toolkit in community-based organizations: a qualitative study
Source: Implement Sci Commun. 2021 May 26;2:55. doi: 10.1186/s43058-021-00158-1 (PMC8157454; doi:10.1186/s43058-021-00158-1)
Supplement: Supplementary file 2 — Additional file 2. End of Pilot Interview Guide. [file 43058_2021_158_MOESM2_ESM.doc]

## Additional File 2. End of Pilot Study Interview Guide

**ACT SMART End of Study Interview Guide**

I will be asking you a few questions today about how feasible, acceptable and useful the ACT SMART toolkit and facilitation meetings were to you, overall.

A. ACT SMART Toolkit

1. How useful was the ACT SMART toolkit? *(probe for website overall & web-based activities for each of these questions)*
   1. How feasible was the ACT SMART toolkit?
   2. How satisfied were you with the ACT SMART toolkit?
2. How challenging was the ACT SMART toolkit? *(probe for website overall & web-based activities)*
   1. Was there anything about your agency/agency site that made it challenging to use?
   2. Was there anything about your implementation team that made it challenging to use?

B. ACT SMART Training Model

1. How useful was the ACT SMART orientation training?
2. What were your thoughts about the facilitation meetings?
3. How challenging were the ACT SMART facilitation meetings?
   1. How difficult was it to complete the facilitation meeting action steps?
   2. How difficult was it for you to schedule and attend the orientation training and facilitation meetings?

C. Impact of ACT SMART on agency

1. How would you know if ACT SMART were successful at your agency site? How would you measure success?
2. Has there been any value to using ACT SMART, to you or your agency site? In what way?
3. What changes have you observed at your agency site since you began using the ACT SMART toolkit?
   1. How has your agency’s process changed when you are thinking about doing something new?
   2. How has your knowledge and skills to adopt new research-based treatments changed?
   3. How have your skills to adapt research-based treatments for use within your agency changed?
   4. How has your ability to identify specific strategies to implement the use of new research-based treatments changed?
   5. How has your ability to use specific strategies to support the use of new treatments changed?

D. Future use of ACT SMART Toolkit

1. Would you be interested in continuing to use ACT SMART at your agency?
2. Would you buy ACT SMART for your agency, if it were for purchase?
3. How much would your agency site be willing to pay, if it were for purchase?

E. Recommendations

1. What changes would you recommend for the ACT SMART toolkit and facilitation meetings? *(Probe for website overall & web-based activities)*
