# Supplementary material for: Facilitators and barriers to the utilization of the ACT SMART Implementation Toolkit in community-based organizations: a qualitative study
Source: Implement Sci Commun. 2021 May 26;2:55. doi: 10.1186/s43058-021-00158-1 (PMC8157454; doi:10.1186/s43058-021-00158-1)
Supplement: Supplementary file 3 — Additional file 3. Codebook. [file 43058_2021_158_MOESM3_ESM.doc]

## Additional File 3. Code book

| **Main Code/Subcode** | **Description/Examples** |
| --- | --- |
| 1. **Facilitators to Use of ACT SMART** | General factors or characteristics facilitating the use of ACT SMART. May be related to factors/activities specific to ACT SMART, that are not phase-specific.  *E.g. ACT SMART is intuitive, functional, straightforward.* |
| 1a. **Website**  1ai. Perceived Ease of Use  1aii. Perceived Usefulness | Factors specifically related to the website that facilitated the use of ACT SMART.  1ai. Refers to comments regarding how easy it was to use the website *(e.g. comments like “user-friendly” or “easy to navigate)*  1aii. Comments about how useful the website was *(e.g. the website contents were really useful/helpful, the resources on the website were useful)* |
| 1b. Facilitation Team | Any responses regarding the FT members or their responsiveness as facilitators to ACT SMART. Includes responses re: FT being responsive to requests from the agency, or needs of the agency, *and* that the responsiveness to these requests/needs were facilitators to ACT SMART |
| 1bi. FT Meetings | Comments specific to the FT meetings.  *E.g. Perceived as useful, scheduling was flexible, agenda prepared ahead of time, provided agency with information, content, ideas* |
| 1c. **Phase Specific Facilitators to Use of ACT SMART**  1ci. Phase 1  1cii. Phase 2  1ciii. Phase 3  1civ. Phase 4  1cv. Phase 5 | Phase specific factors facilitating the use of ACT SMART. This includes any activities specific to a single phase of ACT SMART  *E.g.:*   - *Phase 1: agency assessment (embedded into activities, useful, mapped out next steps, aligned with staff vision, easy to complete)* - *Phase 3: developing training plan was helpful to agency* |
| 1d. **Inner Context Factors as Facilitators to Use of ACT SMART** | Specific inner context factors that facilitated ACT SMART Use (Moulin et al., 2019) |
| 1di. Individual Characteristics | Characteristics of the staff members that facilitate AS use (e.g. previous experience in a related field – e.g. with implementation work, with the EBP, with autism, with research) |
| 1dii. Organizational characteristics | Staff working well together or other comments regarding agency culture that facilitate AS use. |
| 1e. Additional Resources from ACT SMART | Additional resources provided from ACT SMART (e.g. access to other agencies, literature. Do not code if FT provides agency with AS resources that were supposed to be provided- e.g. paper version of budget form) |
| 1. **Barriers to Use of ACT SMART** | General factors hindering the use of ACT SMART. May be related to factors/activities specific to ACT SMART. |
| 2a. **Website Issues**  2ai. Perceived Ease of Use  2aii. Perceived Usefulness | Factors specifically related to the website that impeded the use of ACT SMART. *E.g. tech glitches*  1ai. Refers to comments regarding how hard it was to use the website (e.g. comments like “not user-friendly” or “difficult to navigate” or “not easy to access”, includes comments re: not remembering password)  1aii. Comments about website not being useful/needed (e.g. the website contents were not helpful, the resources on the website were not useful) |
| 2b. Perceived Lack of Resources | Comments regarding the lack of appropriate or helpful resources that IT members believed would be provided from ACT SMART. This should be distinct from resources that come from inside the agency or are specific to the agency, and focus on resources that agencies believed ACT SMART was responsible for providing or did not provide but would have facilitated ACT SMART use.   - *Eg. ACT SMART did not provide access to related journals* |
| 2c. **Phase Specific Barriers**  2ci. Phase 1  2cii. Phase 2  2ciii. Phase 3  2civ. Phase 4  3cv. Phase 5 | Factors specific to a phase that impeded ACT SMART use.  - *Eg. issues with budget worksheet (phase 2)*  Comments re: staff training (the activity) as a barrier to completing ACT SMART, specific to a phase |
| 2d. **Inner Context Factors as Barriers** | Specific inner context factors that impeded ACT SMART Use *E.g. agency members too busy, limited access to resources needed that ACT SMART is not responsible for providing, agency being located far away from AS* |
| 2di. Time Constraints | Comments regarding staff members being too busy or not having time to complete any aspect of AS. |
| 2dii. Scheduling | Comments regarding scheduling being difficult as a barrier to AS. |
| 2diii. Funding | Comments regarding issues related to funding as impeding ability to complete AS (e.g. funding limiting access to EBP materials, thus slowing down AS progression) |
| 2div. Organizational characteristics | Comments regarding the characteristics of the agency that impede ability to complete activities for AS (i.e. productivity expectations, location, IT problems at agency) |
| 2e. ACT SMART not tailored to agencies | Responses discussing ACT SMART being general or not tailored to agencies and their specific needs as a barrier to ACT SMART. |
| 2f. Lack of Responsiveness from FT | Comments from IT members regarding asking FT for resources or giving them feedback about an issue (e.g. website) but not hearing back/issues not getting fixed |
| 1. **General Inner Context Factors** | General inner context factors that neither facilitated/impeded the use of ACT SMART and are not a result of ACT SMART use. May include changes to agency *since* ACT SMART began.  *Examples:.*   - Staff unaware of ACT SMART - Staff excited about ACT SMART - Less turnover - New trainings - At beginning of pilot/already existing   - Consultants felt they didn’t have enough support   - Agency undergoing leadership changes |
| 1. **Barriers to EBP Implementation** | Any comments on factors hindering EBP implementation, including inner context factors |
| 1. **Facilitators to EBP Implementation** | Any comments on factors facilitating EBP implementation, including inner context factors (EBP factors – adaptability & flexibility) |
| 1. **Suggestions to Improve ACT SMART Toolkit** | Comments re: suggestions to improve the toolkit overall |
| 6a. Suggestions to improve FT Meetings | Suggestions to improve facilitation meetings   - Spacing out facilitation meetings - FT meetings to be based on agency needs   - *Higher frequency of FT meetings if needed for that specific phase*   - *FT sends summary to agency instead of in-person meeting or other ways to disseminate information* |
| 6b. Suggestions to improve resources | Suggestions to improve resources -*E.g. access to relevant literature* |
| 6c. Suggestions to improve website | Suggestions to improve website |
| 6d. Phase Specific Suggestions  6di. Phase 1  6dii. Phase 2  6diii. Phase 3  6div. Phase 4  6dv. Phase 5 | Suggestions to improve ACT SMART that are specific to a phase and/or its activities |
